# From pancreas to lungs: The role of immune cells in severe acute pancreatitis and acute lung injury

**DOI:** 10.1002/iid3.1351

**Published:** 2024-07-18

**Authors:** Qi Liu, Xiaomei Zhu, Shubin Guo

**Affiliations:** ^1^ Emergency Medicine Clinical Research Center, Beijing Chao‐Yang Hospital Capital Medical University Beijing China; ^2^ Beijing Key Laboratory of Cardiopulmonary Cerebral Resuscitation Beijing China

**Keywords:** acute lung injury, cytokines, immune cells, immunomodulation therapy, macrophages, neutrophils, severe acute pancreatitis, T cells

## Abstract

**Background:**

Severe acute pancreatitis (SAP) is a potentially lethal inflammatory pancreatitis condition that is usually linked to multiple organ failure. When it comes to SAP, the lung is the main organ that is frequently involved. Many SAP patients experience respiratory failure following an acute lung injury (ALI). Clinicians provide insufficient care for compounded ALI since the underlying pathophysiology is unknown. The mortality rate of SAP patients is severely impacted by it.

**Objective:**

The study aims to provide insight into immune cells, specifically their roles and modifications during SAP and ALI, through a comprehensive literature review. The emphasis is on immune cells as a therapeutic approach for treating SAP and ALI.

**Findings:**

Immune cells play an important role in the complicated pathophysiology ofSAP and ALI by maintaining the right balance of pro‐ and anti‐inflammatory responses. Immunomodulatory drugs now in the market have low thepeutic efficacy because they selectively target one immune cell while ignoring immune cell interactions. Accurate management of dysregulated immune responses is necessary. A critical initial step is precisely characterizing the activity of the immune cells during SAP and ALI.

**Conclusion:**

Given the increasing incidence of SAP, immunotherapy is emerging as a potential treatment option for these patients. Interactions among immune cells improve our understanding of the intricacy of concurrent ALI in SAP patients. Acquiring expertise in these domains will stimulate the development of innovative immunomodulation therapies that will improve the outlook for patients with SAP and ALI.

## Graphical abstract

The article attentively demonstrates how immune cells serve as a “bridge” between severe acute pancreatitis and acute lung damage. The immunologic properties of immune cells may result in better patient outcomes and the creation of novel immunotherapies. Immune cells can also be used to predict the course of a disease, giving doctors a strong starting point.

## 1 INTRODUCTION

Severe acute pancreatitis (SAP) is a serious inflammatory disease of the pancreas. In its early phases, SAP may be accompanied by multiorgan dysfunction syndrome (MODS) and systemic inflammatory response syndrome (SIRS).^1^ The mortality rate can reach up to 30%.^2^, ^3^ For this reason, patients with SAP typically require intensive care unit treatment. Following discharge, patients with SAP have a significantly decreased life expectancy, and some may experience uncontrollable aftereffects such as pancreatic pseudocyst, extrapancreatic dysfunction, diabetes mellitus, and chronic pancreatitis (CP).^4^, ^5^, ^6^, ^7^

The lung is the organ most frequently affected in patients with SAP, aside from the pancreas. Roughly one‐third of SAP patients will develop acute lung injury (ALI) or acute respiratory distress syndrome (ARDS) in severe cases.^8^, ^9^ Based on statistical data, approximately 10% of patients with SAP and ALI will die.^10^, ^11^

**Figure 1:**
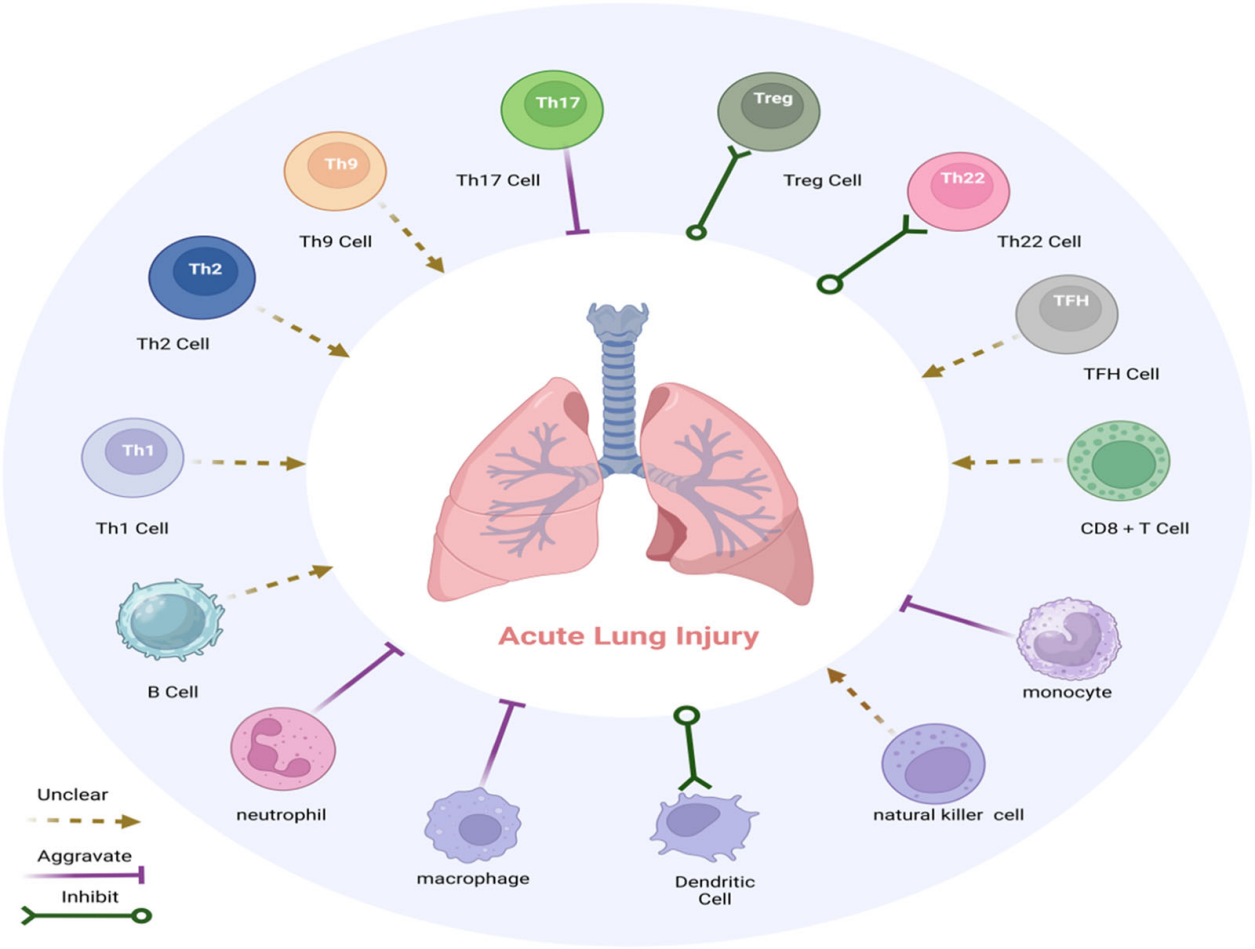
Role of immune cells in SAP‐associated ALI. Regulatory T (Treg) cell, T helper (Th) cell 22, and dendritic cells play a protective role and attenuate lung injury. Th17, macrophage, neutrophil, and monocyte exacerbate lung injury. The exact role of natural killer (NK) cell, B cell, CD8^+^T cell, Th1, Th2, Th9, and T follicular helper (TFH) in lung injury is unknown. ALI, acute lung injury; SAP, severe acute pancreatitis.

However, in contrast to direct lung injury caused by pulmonary pathogen infection, SAP patients often have indirect lung injury, which is a totally different syndrome. Indirect lung damage is frequently caused by systemic inflammation, which draws active immune cells to the lungs to carry out their immunological effects. This eventually leads to lung epithelial cell death and impaired lung barrier function.^12^, ^13^ Figure 1 illustrates the function of immune cells during ALI. But eventually, lung infections also strike certain SAP‐ALI patients. When SAP patients present with pulmonary symptoms, clinicians must evaluate the patient's lungs, as well as chest imaging and sputum etiology, and adjust the treatment regimen as soon as possible. Due to the complexity of body immunity, the unknown etiology of ALI in these patients, and the inability of physicians to implement specific targeted therapy for the pathogenesis, ALI continues to be the primary cause of death for SAP patients.^14^, ^15^, ^16^

Recent studies have shown that an imbalance in immune control is at the root of SAP. SIRS and compensatory anti‐inflammatory response syndrome (CARS) cohabit and alternate throughout the complex SAP course. The same immune cells or inflammatory chemicals also have pro‐ or anti‐inflammatory actions at different stages and pathological states of SAP. Whether the pro‐ or anti‐inflammatory response predominates, the SAP patient's condition deteriorates when the pro‐/anti‐inflammatory equilibrium is broken.

Immunomodulation therapy provides new options for treating SAP sufferers. After precisely regulating the inflammatory response and restoring the body's immune balance, it reduces damage to related organs and improves prognosis. Immune cells cooperate to regulate one another rather than acting alone since the immune system is a complex one. Regrettably, the present immune regulation strategies frequently influence one kind of immune cell or cytokine and have negligible effect.

We forecast the creation of innovative medicines that target a wide range of immune cells and cytokines. The article summarizes the role of immune cells in the development of SAP and ALI, detailing the linkages between immune cells. We believe that clinicians and researchers will benefit from this fresh perspective.

## 2 NEUTROPHILS

The activation of the immune system is a crucial phase in the transition from AP to SAP, as it can result in SIRS and MODS.^17^ Neutrophils are the first immune cells to be attracted to the site of pancreatic damage, and they initiate the inflammatory response to eliminate necrotic tissue. Neutrophils are stimulated by released cytokines.^18^ On the one hand, activated neutrophils release numerous cytokines that trigger inflammatory reactions both locally and systemically.^19^ Meanwhile, they utilize a variety of defense mechanisms, including phagocytosis, the production of reactive oxygen species (ROS), and degranulation to create neutrophil extracellular traps (NETs), to thwart the invasion of pathogens.^20^

Since our understanding of neutrophils' significance has grown, researchers have learned that they are remarkably malleable. Neutrophils can modify their phenotypic and function in response to changes in the microenvironment. Different neutrophil phenotypes have different roles in the development and control of inflammation. The roles that different neutrophil phenotypes play in the pathophysiology of SAP and ALI will be covered in this section, with a focus on the mechanisms by which NETs worsen both disorders.^21^, ^22^

### 2.1 The dynamics of neutrophil phenotype

Neutrophils are remarkably adaptable. Neutrophil phenotype and function vary dynamically in response to microenvironmental changes. In the field of tumor research, such as breast cancer,^23^ pancreatic cancer,^24^ and lung cancer,^25^ neutrophils were initially defined as two phenotypes: neutrophil type 1, or anti‐tumourigenic N1, which exerts a tumor‐suppressor role, and neutrophil type N2, or protumorigenic neutrophils, which promotes tumorigenesis.^26^ Neutrophils have subsequently been classified as proinflammatory developmental type N1 and anti‐inflammatory type N2, depending on the type of cytokines they release.^27^ N1 neutrophils generate proinflammatory cytokines such as tumor necrosis factor (TNF)‐α and interleukin (IL)‐2 and express intercellular adhesion molecule (ICAM)‐1 and Fas abundantly. N2 neutrophils, on the other hand, produce considerable amounts of arginase. Circulating arginine depletion affects T lymphocyte proliferation.^28^ N1 neutrophils may convert into N2 neutrophils in response to lipopolysaccharide (LPS), interferon (IFN)‐γ, and other stimuli, while N2 neutrophils can change in phenotype in response to transforming growth factor‐β (TGF‐β), IL‐10, and l‐lactate.^29^

The current focus of research on the association between neutrophil phenotype and sickness has been aseptic necrosis, which includes myocardial infarction (MI) and ischemic stroke. According to earlier research by Ma et al.,^30^ neutrophils in the heart showed two phenotypes, N1 and N2, when MI started. On the first day after MI, neutrophils predominantly exhibited the N1 phenotype, with a small amount of N2 phenotype also visible. The anti‐inflammatory N2 type then gradually took dominance as the MI worsened, possibly due to tissue repair and inflammation decrease. In an ischemic stroke, neutrophils function as both “injury” and “protection.” N1 neutrophils release proinflammatory molecules and ROS during the early stages of ischemic stroke, which causes brain tissue to be destroyed. Later on, N2 neutrophils gradually increase and contribute to neuroprotection.^31^ According to the findings mentioned above, the alteration in neutrophil phenotype is strongly associated with how the illness progresses, and targeting N1 or N2 neutrophils specifically may lead to the development of new therapeutic approaches.

Unfortunately, there aren't many rigorously scientific investigations to look into how the neutrophil phenotype varies in SAP and ALI. It is plausible to conjecture, nevertheless, that patients suffering from SAP and ALI also experience these modifications in neutrophil phenotype. Proposing therapeutic options to target neutrophil phenotypic changes will expand the possibilities for treating SAP and ALI.

### 2.2 NETs exacerbate pancreatic injury

As research on NETs continued, it was discovered that NETs are complex in composition and can cause a variety of inflammatory reactions. Researchers discovered that neutrophil‐produced NETs exacerbated pancreatic tissue injury and lung injury in the pancreatic tissue of the SAP mice.^22^, ^32^ The extracellular reticular fibrous structure known as NETS is composed of cytosolic and granule proteins that are arranged on fragmented chromatin (DNA) and mixed with other proteins such as peptidoglycan binding protein, neutrophil elastase (NE), cathepsin G (CG), myeloperoxidase (MPO), and others. Lethality and antibacterial activity are strongly linked to its ingredients.^33^

One important component found in pancreatic acinar cells is called signal transducer and activator of transcription (STAT)−3, and it plays a dual role in initiating and escalating the inflammatory response. NETs promote the activation of STAT‐3 in acinar cells. The activation of the Janus kinase/STAT (JAK/STAT) pathway leads to the destruction of endothelial cells and intensifies the inflammatory response by inducing the expression of ICAM‐1.^15^

Trypsin can be inappropriately activated by neutrophil‐produced NTEs,^34^ primarily through an increase in matrix metalloproteinase (MMP)‐9.^35^ MMP‐9 is the most extensively researched member of a class of endopeptidases known as MMPs, which can degrade matrix proteins as well as other nonmatrix targets such as cytokines and chemokines. MMP‐9, as a trypsinogen activator, can stimulate trypsin activity and induce tissue injury.^36^ Merza et al.^32^ discovered that taurocholate‐induced acute pancreatitis mice resulted in a significantly higher quantity of NETs in their plasma, along with an approximately a roughly eightfold increase in MMP‐9. When AP mice were given deoxyribonuclease I (DNase I) to inhibit NETs, the plasma MMP‐9 levels decreased by 93%, and there was also less damage to the pancreas.

NETs frequently play a dual role in SAP. In a protective role, moderation in the release of NETs can lead to their accumulation and the formation of a physical barrier, such as walled‐off necrosis or a pancreatic pseudocyst. This barrier separates normal from aberrant tissue in space and inhibits the spread of infections and inflammatory agents.^37^

However, excessive release of NETs may lead to tissue damage and malfunction. This was also validated by Leppkes et al.^38^ The alkaline environment of pancreatic juice promotes the formation of NETs, and the accumulation of NETs in the pancreatic duct leads to pancreatic duct obstruction and pancreatic secretion to be inhibited. When NETs are deposited in pancreatic acinar cells, the particular cytotoxic effect of histones causes damage to the serous membrane of pancreatic acinar cells. This leads to leakage of acinar cytoplasm and, ultimately, death of pancreatic acinar cells.^22^

Additionally, NETs modulate blood flow. Histone proteins are primarily responsible for thrombosis, which is promoted by the deposition of NETs in blood vessels. Histones stimulate vascular endothelial cells to release von Willebrand factor (vWF), enabling platelets to aggregate on the reticular fibrous skeleton of NETs and initiate thrombosis.^39^, ^40^ Furthermore, the free DNA generated by NETs has a notable procoagulant effect. Liaw et al.^41^ observed that the free DNA of NETs may initiate blood coagulation, which is dependent on both factor xi (FXI) and FXII. NETs are the principal cause of disseminated intravascular coagulation in SAP.

### 2.3 NETs exacerbate lung injury

**Figure 2:**
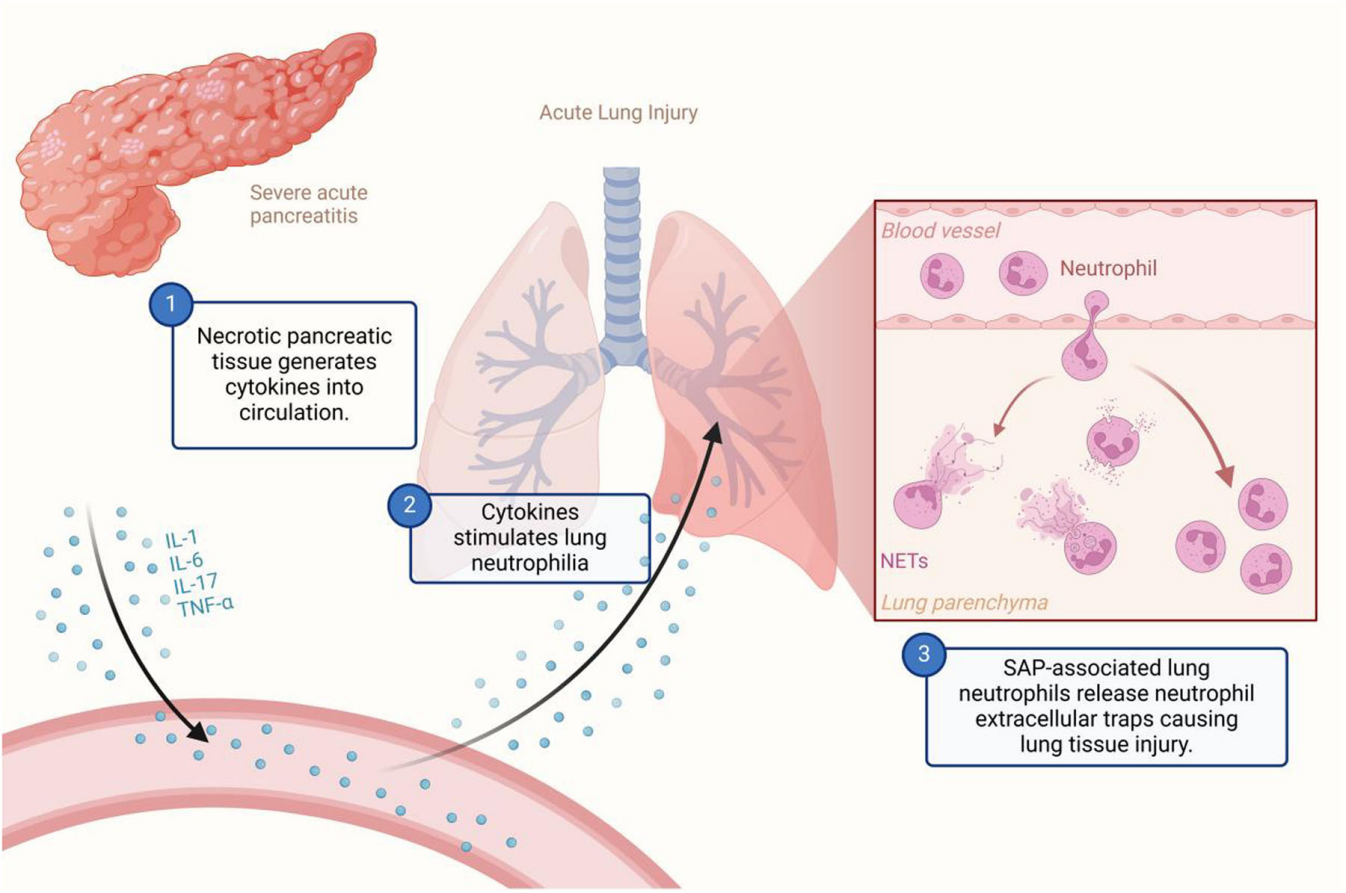
Neutrophils release NETs, leading to lung injury. Necrotic pancreatic cells generate and release a multitude of cytokines, including IL‐1, IL‐6, TNF‐α, and others, into the circulation. Large amounts of cytokines accumulate as they circulate in the lungs. A large number of circulating neutrophils are drawn into the lungs, where they release NETs and cause damage to lung tissue. NETs, neutrophil extracellular traps; TNF‐α, tumor necrosis factor.

In response to inflammatory stimuli and chemokines, a substantial number of neutrophils migrate to the lung tissue through blood circulation, releasing NETs. This is believed to be a characteristic feature of SAP‐ALI (Figure 2). A significant amount of NETs will be deposited in lung tissue and alveolar cavities when the production rate of NETs exceeds its breakdown rate. This can lead to acute lung damage and, in extreme situations, the development of ARDS in patients.^42^ Cortjens et al.^43^ reported that children infected with respiratory syncytial virus had substantial NTEs formation in bronchoalveolar lavage fluid and lung tissue sections. NETs have also been observed in mucus plugs that obstruct airways.

Relevant literature reports have shown that NETs can cause excessive mucin production by airway epithelial cells, leading to an increase in the viscosity of airway tissues. They can also interfere with the oscillation frequency of the mucosal cilia and weaken the airway's ability to clean itself, which makes it difficult for sputum clots to be expelled and ultimately leads to airway obstruction and infection.^44^, ^45^

Due to the unique characteristics of NETs, lung epithelial cells can also be injured by NETs through histone proteins. This can lead to microthrombosis, intra‐alveolar hemorrhage, and alveolar epithelial cell vacuolization.^46^ NE mostly exhibits the proteolytic impact of NETs.^47^ NE, the most abundant and potent proteolytic enzyme in NETs, promotes lung tissue injury through several different mechanisms. The permeability of alveolar capillaries is closely associated with NE. By hydrolyzing E‐cadherin, NE disrupts intercellular connections and induces pulmonary microvascular damage.^48^, ^49^, ^50^ Additionally, NE can degrade heparan sulfate proteoglycans (HSPG) in the endothelial cell matrix and reduce the synthesis of endothelial glycocalyx (eGCX).^51^ eGCX, composed of HSPG and hyaluronic acid, adheres to the vascular endothelium and regulates vascular permeability and tension, contributing to homeostasis.^52^ Reduced eGCX production and increased vascular permeability seem to account for the high incidence of pleural effusion in SAP patients.

## 3 MACROPHAGES

Due to their heterogeneity, macrophages can respond to variations in their microenvironment by adjusting their states and functions.^53^ In early SAP, activation of the NF‐κB inflammatory signaling pathway damages acinar cells.^54^, ^55^ Necrotic acinar cells produce damage‐associated molecular patterns (DAMPs). In a process dependent on Toll‐like receptors (TLRs), macrophages respond to DAMPs and differentiate into M1‐classically activated macrophages. M1 macrophages remove necrotic tissue and release inflammatory mediators such as TNF‐α, IL‐1β, IFN‐γ, and IL‐6.

**Figure 3:**
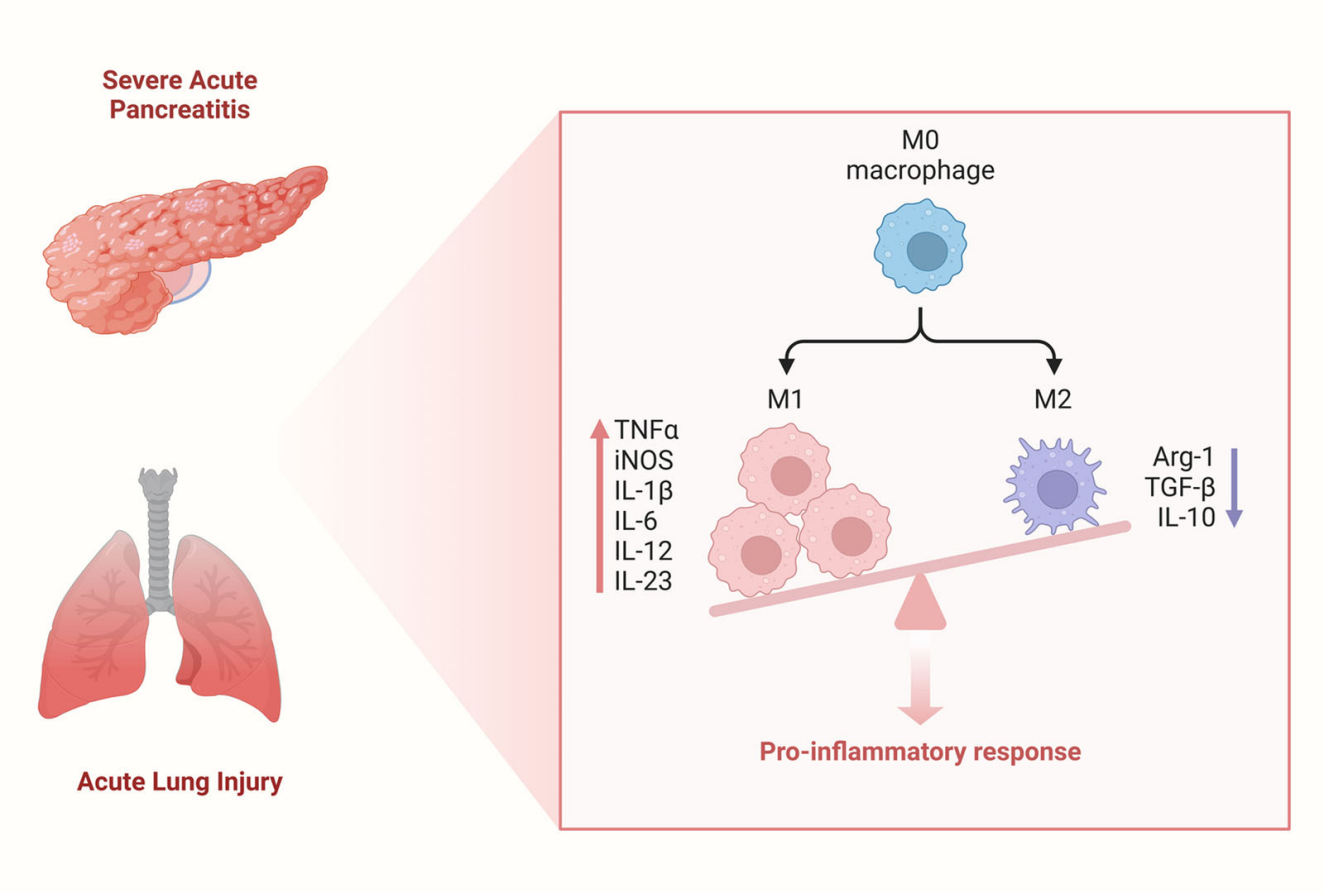
Abnormal macrophage differentiation leads to SAP and ALI. SAP and ALI are the results of abnormal macrophage differentiation. M1 macrophages were predominant, while M2 macrophages were inhibited. Primarily, M1 cells exhibit proinflammatory properties and produce abundant proinflammatory cytokines, including IL‐1β, IL‐6, IL‐12, and TNF‐α, which circulate into lung tissue and worsen inflammation there. Inversely, M2 macrophages release cytokines such as IL‐10 and TGF‐β to reduce inflammation and alleviate damaged tissues. ALI, acute lung injury; SAP, severe acute pancreatitis; TGF‐β, transforming growth factor‐β; TNF‐α, tumor necrosis factor.

As the disease progresses, macrophages differentiate into M2‐alternatively activated macrophages, which release cytokines such as IL‐10 and TGF‐β to regulate inflammation. M2 macrophages play an indirect role in pancreatic tissue regeneration due to their anti‐inflammatory properties.^56^, ^57^ Since M1 and M2 macrophages are hostile, it may be quite beneficial to balance their ratios to prevent the development of SAP and ALI^58^ (Figure 3).

### 3.1 Macrophage differentiation

Pancreatic cells eventually necrose and release DAMPs, including high‐mobility group box‐1 (HMGB1), heat shock proteins, S100 proteins, and others, when the inflammation worsens. These DAMPs have been found to accelerate the evolution of aseptic inflammation in the pancreas.^59^, ^60^, ^61^, ^62^ DAMPs are commonly found inside tissue cells. However, upon tissue damage, DAMPs are discharged extracellularly. These DAMPs have the same pattern recognition receptors (PRRs) as pathogenic microorganisms. Sterile inflammation is the term for the inflammatory response that is triggered solely by DAMPs in the absence of pathogenic germs. The goal of sterile inflammation is to reduce inflammation rather than eradicate the pathogen.^63^ One such instance of sterile inflammation that is primarily brought on by DAMPs is the early stages of acute pancreatitis.^64^

When DAMPs bind to PRRs, certain signaling pathways are triggered, which regulate the partitioning of macrophages.^65^ TLRs are the most common type of PRR, with 11 functioning TLRs found in the human body.^66^ TLRs, which are type I transmembrane proteins with three main structural regions, are found on the surface of immune cells like macrophages, dendritic cells (DCs), and lymphocytes, as well as in a wide range of organs like the liver, kidney, pancreas, and intestinal tissues.^67^ The direction of macrophage differentiation is controlled by DAMPs generated by pancreatic cell necrosis, which bind to and recognize TLRs on macrophage cell membranes.

DAMPs bind specifically to TLRs, regulating macrophage differentiation. Of all the DAMPs that promote the development of pancreatitis, HMGB1 is the most representative one. HMGB1 is a nuclear DNAbinding protein that is released into the extracellular milieu when cells apoptosis or necrosis, and it is an important DAMP in the advancement of SAP.^68^ During SAP, HMGB1 is markedly elevated, and the degree of the rise corresponds with the severity of SAP.^69^ Qu et al.^70^ reported that HMGB1 is closely associated with ALI and can be used as a biomarker for prognosis.

Indeed, when TLR4 on the macrophage cell membrane recognizes HMGB1 released into the extracellular environment, it is activated instantly. Then, intracellular signaling molecules such as interleukin‐1 receptor‐associated kinases (IRAKs), tumor necrosis factor receptor‐associated factors (TRAFs), and other intracellular signaling molecules are activated by TLR4 through the MyD88‐dependent pathway. Intracellular signaling cascade amplification results in the activation and ectopic nuclear factor NF‐κB. This, in turn, causes the transcription and translation of inflammatory cytokines, leading to the eventual release of copious amounts of inflammatory mediators, including IL‐6 and TNF‐α.^71^, ^72^, ^73^ Through a TLR4‐mediated route, HMGB1 stimulates macrophage reprogramming toward an M1 and the generation of proinflammatory cytokines linked to the M1, which affects M2 polarization.^74^, ^75^

### 3.2 Macrophages in SAP

While coculturing macrophages and live acinar cells in vitro, Sendler et al. noticed trypsin activity.^76^ It was eventually found that there appeared to be a significant increase in trypsin activity. Once drugs suppressed macrophages, trypsinogen activity decreased. There is a potential link between trypsinogen activation and macrophages.

Indeed, macrophages surrounding pancreatic tissue phagocytize a significant amount of trypsinogen along with necrotic cells. These phagocytosed zymogens are converted to trypsin by the lysosomal hydrolase cathepsin B (CTSB) through a process similar to trypsinogen activation in acinar cells.^77^ This appears to be the reason why trypsin activity variations in AP patients exhibit two peaks.

Macrophages activate trypsinogen and release proinflammatory chemicals, including TNF‐α, IL‐6, and IL‐1, leading to increased inflammation.^78^, ^79^ According to earlier studies, macrophage LPS activates p38α, which in turn initiates the downstream cascade of TNF‐α, IL‐6, and IL‐1.^80^, ^81^ Inhibiting p38α activity can effectively reduce inflammatory responses. Fan et al.^82^ reported that the macrophage‐derived p38α inhibitor SB202580 reduced pancreatic tissue damage in mice and decreased mRNA expression of cytokines TNF‐α, IL‐6, and IL‐1.

Some individuals may develop pancreatitis‐associated ascitic fluid (PAAF), which contains high levels of trypsin and inflammatory chemicals as the disease progresses. In this scenario, peritoneal macrophages will differentiate into M1 macrophages, exacerbating the inflammatory response.^83^, ^84^

The portal vein carries trypsin and inflammatory mediators to the liver, causing Kupffer cells in the hepatic sinusoids to differentiate into several phenotypes.^85^ Differentiated Kupffer cells are responsible for the majority of circulating inflammatory factors.^86^ Mice's lung tissue damage decreased after cobalt chloride (GdCl_3_) was used to eliminate Kupffer cells, while pancreatic tissue damage remained relatively the same.^87^ The conclusion is that Kupffer cells play a crucial role in enhancing the inflammatory response and triggering distant organs during SAP, although they have little effect on the pancreas itself. This provides a possible explanation for the mild systemic symptoms experienced by pancreas transplant patients with SAP.^88^ The majority of the pancreatic venous blood flows back to the systemic circulation, not the liver. Vascular remodeling is often involved in pancreas transplantation.

Exosomes are extracellular vesicles with a communicative function that influences cell activation and proliferation. They often resemble recognized spherical bubbles and contain a variety of substances, including proteins, lipids, DNA, and RNA.^89^ Alesanco et al. discovered exosomes in peripheral blood and PAAF in mice with acute pancreatitis.^90^ Following labeling with PKH26, exosome vesicles were injected into the veins of mice and monitored. Ultimately, research revealed that Kupffer cells and hepatocytes gathered and removed the exosomes. Afterward, these cells released exosomes produced by the liver into the plasma, which were then carried throughout the body and contributed to the damage of distant organs.^91^

### 3.3 Macrophages in ALI

When it comes to ALI, macrophages are crucial.^92^ SAP promotes the release of trypsin and inflammatory mediators into the circulation. Activation of Kupffer cells further increases the release of these mediators. In response to stimulation, lung macrophages may differentiate into two distinct phenotypes: M1 macrophages and M2 macrophages. The lung is composed of three types of macrophages: alveolar macrophages (AMs), pulmonary intravascular macrophages (PIMs), and interstitial macrophages (IMs). The three types of macrophages induce different levels of lung damage.^93^

Located on the surface of the alveolar cavity, AMs are the most abundant immune cells in the lung and play a crucial role in initiating role during ALI. Diffuse alveolar damage and exudation occur during the early stages of ALI since AMs differentiate into the M1 phenotype in response to circulating inflammatory mediators. Subsequently, they continually secrete inflammatory factors, including TNF‐α, IL‐6, IL‐8, NO, and ROS.^94^, ^95^ At the same time, AMs can recruit neutrophils, monocytes, T lymphocytes, and other immune cells to the lungs, contributing to pulmonary vascular endothelial cell injury and exacerbating pulmonary edema.^96^

It has been demonstrated that lung tissue injury arises from specific levels of PIM in alveolar septal capillaries. Reducing PIM levels decreases the amounts of proinflammatory mediators, thereby limiting lung tissue damage.^97^ It is still unclear why PIM promotes lung injury, and further investigation will be required to determine this mechanism.

Lung macrophages underwent a phenotypic conversion from M1 to M2 after treatment and intervention, alleviating the condition of ALI. Ding et al.^98^ revealed that Adropin enhances macrophage differentiation into M2‐type macrophages through PPARγ phosphorylation. M2 macrophages can repair lung tissue and protect alveolar epithelial cells by enhancing the production of IL‐10, TGF‐β, and insulin‐like growth factor.^99^ After controlling ion channels and releasing excess fluid, M2 macrophages can help improve ventilation and reduce pulmonary edema.^100^ However, fibrosis, which is caused by substantial collagen deposition in alveolar epithelial cells, occurs in ALI patients when M2 macrophages become over‐polarized.^92^, ^101^ As a result, the balance between M1/M2 macrophages regulates the severity of lung damage. Patients may benefit from interventions targeting macrophages.

Certainly, macrophage pyroptosis may lead to lung tissue injury. Pyroptosis is a form of caspase‐1‐dependent programmed cell death associated with inflammasome activation.^102^ Wu et al.^103^ pointed out that the NLRP3 inflammasome is crucial for macrophage pyroptosis. Activation of the NLRP3 inflammasome triggers caspase 1 maturation, resulting in pyroptosis in macrophages. Pyroptosis ruptures the cell membrane and releases proinflammatory substances into the environment, leading to local or systemic inflammation.^104^ The NLRP3 inhibitor can significantly reduce lung damage.^105^ NLRP3 could serve as an intervention target to reduce lung injury and inflammation.^106^, ^107^ Moreover, neutrophil migration driven by macrophage pyroptosis could exacerbate lung tissue injury.^108^

## 4 OTHER INNATE IMMUNE CELLS

### 4.1 DCs

DCs are derived from bone marrow hematopoietic cells and contain two major subpopulations: monocytic DCs (mDCs) and plasmacytoid DCs.^109^ As innate immune cells, DCs phagocytose and eliminate infections and other toxic chemicals. Furthermore, DCs operate as antigen‐presenting cells, activating T and B lymphocytes to participate in adaptive immunity.^110^

Bedrosian et al.^111^ discovered that DCs have a protective impact on acute pancreatitis. In contrast, Xu et al.'s investigation^112^ came to a different conclusion. It was shown that when DCs were activated in the pancreas of AP rats, local Th1 and Th17 were promoted to have proinflammatory effects, whereas Treg was blocked from having anti‐inflammatory effects. Consequently, the pancreatic injury became more severe. It is currently thought that the existence of DCs, particularly pDCs, lessens the severity of harm in cases of ALI. After the DCs were depleted, the degree of lung damage was significantly increased. This could be associated with the fact that lung monocyte chemotactic precursor monocyte chemotactic protein‐1 is regulated by pDCs, which prevents monocyte recruitment to the lung.^13^ In conclusion, more investigation and research are still needed to fully understand the role of DCs in SAP and ALI.

### 4.2 Natural killer (NK) cells

NK cells, which originate from hematopoietic cells in the bone marrow, play a vital role in innate immunity. Scientists have been fascinated by their extraordinary capacity to lyse tumor cells, and a large portion of ongoing research focuses on the part NK cells play in the growth of tumors.^113^ NK cells have recently been linked to pancreatic ductal carcinoma and acute pancreatitis, two disorders connected to the pancreas.

A prospective study comprising 101 healthy participants and 102 patients with pancreatic ductal carcinoma revealed that the overall number of NK cells in cancer patients was substantially lower than in the healthy population and that the activity of NK cells would further decline with the growth of the tumor.^114^ Shifrin et al.^115^ observed that NK cells constantly invaded pancreatic organs in adenovirus‐induced AP mice, peaking in NK content on Day 4 and then declining in quantity until Day 28. NK cells appear to have a role in the development of AP disease, even if the exact mechanisms driving this alteration in NK cells remain unknown. Wei et al.^116^ observed the NK cell alterations in the peripheral blood of 115 MSAP patients and 16 SAP patients. The results revealed that the peripheral hematoma NK cell content was significantly lower in all pancreatitis patients than in healthy controls (HCs), and the NK cell content was positively linked with amylase and lipase activities. This indicates that NK cells could be used in conjunction with amylase and other tests to identify MSAP and SAP at an early stage.

Only the lung parenchyma contains lung NK cells, a form of tissue‐resident NK cell absent from extrapulmonary organs.^117^ In contrast to NK cells in the peripheral circulation, lung NK cells have a lower reactivity to hypothetical cells, which could be beneficial for preserving lung homeostasis. However, further experimental evidence is required to support this.^118^ In conclusion, the hypothesis about lung NK cells offers a fresh viewpoint on studies on SAP‐ALI.

### 4.3 Monocytes

Monocytes are an important component of the body's innate immune defense, playing a role in SAP and ALI. Dabrowski et al.^119^ dynamically assessed peripheral blood monocytes in 35 patients with AP (20 with MSAP and 15 with SAP). According to the study, monocytes underwent considerable activation during AP. On the first day after diagnosis, there was a large and continuous rise in circulating monocytes in both MSAP and SAP patients, particularly SAP patients. On Day 10, the monocyte count peaked and then reverted to normal by Day 30. Changes in peripheral blood monocytes indicate the progression of AP.

ALI develops partly as a result of the elevation in monocyte content. When AP occurs, inflammatory stimuli trigger the release of bone marrow monocytes into the bloodstream, where they take part in the inflammatory response. Specifically, monocytes did not go from the bloodstream into the lung tissues right away; instead, they took 3 days to infiltrate the interior of the lung tissues.

Regretfully, these released monocytes did not show any adaptive change in their cellular flexibility even though they had grown significantly in size. These monocytes clog the pulmonary microvasculature, reducing microvascular blood flow and eventually causing ALI.^120^ Patients' oxygenation levels and lung function improved considerably after monocyte reduction with anti‐CCR‐2 antibodies. A monocyte‐targeted therapy may reverse the consequences of ALI in SAP patients.

## 5 T CELLS

**Figure 4:**
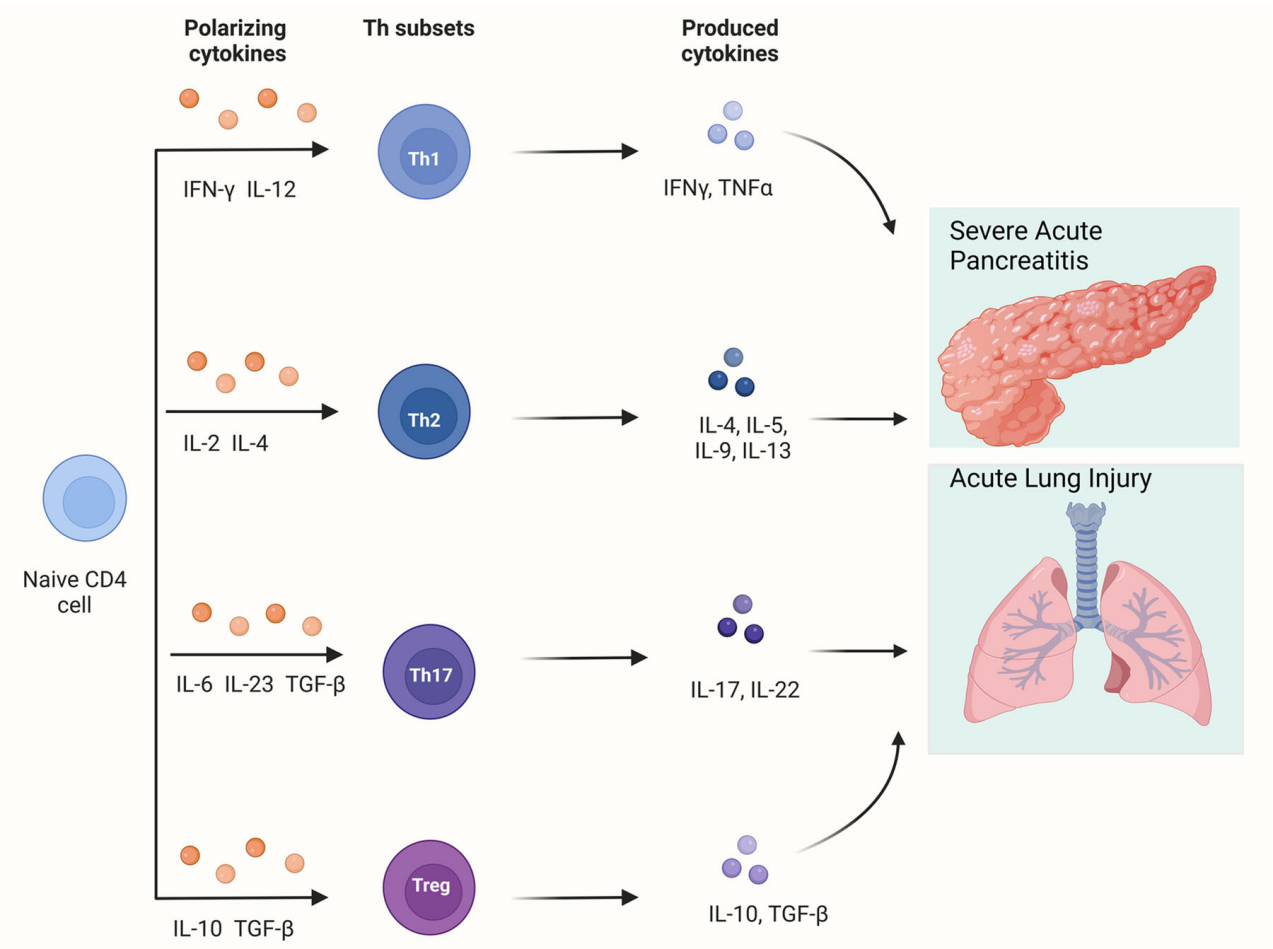
T cell alterations in SAP and ALI. In response to cytokines, naive T cells differentiate into various subsets. Th1‐related cytokines, such as TNF‐α and IFN‐γ, are positively correlated with SAP prognosis. Mild to moderate acute pancreatitis is associated with Th2‐related cytokines, such as IL‐4 and IL‐13. Th17 cells release IL‐17, which exacerbates lung damage and inflammation in the pancreas. Tregs alleviate inflammation through IL‐10 and TGF‐β. In certain circumstances, Treg and Th17 can transform into each other. ALI, acute lung injury; IFN, interferon; SAP, severe acute pancreatitis; TGF‐β, transforming growth factor‐β; TNF‐α, tumor necrosis factor.

Adaptive immune cells, like innate immune cells, play a significant role in the development of SAP.^121^ Based on surface markers and function, adaptive immune cells can be further classified as T cells or B cells.^122^ As the role of B cells in the mechanisms underlying SAP and ALI remains unclear, this section will focus on the role of T cells in SAP and ALI (Figure 4).

When it comes to immunological homeostasis, T cells are crucial.^123^ T cell counts fluctuate during AP development. Furthermore, T cells can also differentiate into a range of subsets with distinct functions, such as CD4 + T helper (Th) cells, regulatory T cells (Treg), and others.^124^ The development of SAP is heavily influenced by T cell counts and subsets.^17^, ^125^

Researchers noticed a decline in the quantity of circulating T cells, specifically CD4 + T lymphocytes, in the early stages of SAP.^125^ Apoptosis occurs in T cells due to abnormal amplification of the Fas/FasL signaling pathway. T‐cell homing to inflamed regions can also lead to a decrease in T lymphocyte count. These two pathways seem to account for variations in the quantity of circulating T cells.^126^, ^127^

In patients with AP, the CD4 + T lymphocyte count may be an indicator of impending disease.^128^ A combined analysis of the number of circulating T cells and organ failure revealed that a decrease in the number of circulating T cells (particularly CD4 + T lymphocytes) was highly associated with organ failure in AP patients.^129^ Moreover, subsets of T cells fluctuate dynamically. There is further evidence that the development of SAP is influenced by the balance of Th1/Th2 and Th17/Treg.^130^, ^131^

### 5.1 CD8+ T cells

Cytotoxic T lymphocytes, or CD8^+^ T cells, primarily destroy intracellular bacteria, viruses, and tumor cells through cell‐to‐cell contacts or the secretion of granzyme and perforin.^132^ Additionally, anti‐inflammatory cytokines, including IL‐10 and TGF‐β, are released to reduce inflammation.^133^ It is critical for maintaining immunological homeostasis. Regarding the modifications of CD8^+^ T cells in SAP patients, there is disagreement. Dabrowski et al.^119^ discovered a decrease in circulating CD8^+^ T cells in patients with SAP. However, Shen et al.^134^ demonstrated an abnormal increase in circulating CD8^+^ T cells. This dynamic variation in CD8^+^ T cells reflects the complexities of the immune system.

### 5.2 Th1/Th2 cells

Th1 cells can promote inflammation by secreting IFN‐γ, which activates macrophages and induces them to differentiate into the M1 phenotype.^135^, ^136^ Th2 cells are known to release cytokines, such as IL‐4, IL‐5, and IL‐13, which are important in the battle against parasite infection and are responsible for controlling different immune cells, such as eosinophils and basophils.^137^ Patients with SAP have been shown to have altered Th1/Th2 cell dynamics. In the early stages of SAP, the Th1 cell count in the blood decreases rapidly while the Th2 cell count increases quickly, leading to a continuous decrease in the Th1/Th2 ratio. However, on Day 14, the Th1/Th2 ratio began to increase.^17^, ^138^, ^139^

Rodriguez et al.^137^ analyzed the relationship between the dynamic modifications of Th1/Th2 cells and the progression of SAP. The Th1 cytokine profile was ultimately found to be highly correlated with the course and outcome of SAP, whereas the Th2 cytokine profile was linked to MAP and MSAP. According to the study, serum levels of IFN‐γ, TNF‐α, IL‐6, and other components were significantly higher in patients with SAP, and Th1 cells were also highly activated. The MSAP and MAP groups exhibited higher levels of Th2‐related cytokines, such as IL‐4 and IL‐13. Preserving the dynamic equilibrium between Th1/Th2 cells has the potential to halt the progression of SAP and improve the prognosis for individuals with SAP.

### 5.3 Th9 cells

Th9 cells, like Th2 cells, are a newly found subset of CD4 + T cells dedicated to generating the cytokine IL‐9.^140^ There has been less investigation on Th9 cell function in AP. Xiao et al.^141^ report that Th9 cells appear to mediate trypsinogen activation. Mogroside IIE administered in vitro reduced pancreatic damage by inhibiting the IL‐9/IL‐9R (IL‐9 receptor) pathway. However, in a pig model of acute edematous pancreatitis and necrotizing pancreatitis, researchers found no appreciable change in IL‐9 levels relative to controls.^142^ Therefore, further investigation is still needed to elucidate the potential role of Th9 cells in the pathophysiology of AP.

### 5.4 Th17 cells

Previous studies have revealed the crucial role that Th17 cells play in SAP.^143^ Th17 cells are strongly associated with the prognosis of SAP patients owing to their proinflammatory characteristics. They can be used as a prognostic marker to guide clinical treatment.^143^, ^144^ During AP, naive CD4 + T cells are recruited into the injured pancreatic tissue due to TGF‐β, IL‐6, IL‐1, and other inflammatory cytokines. After differentiating from recruited CD4 + T cells, Th17 cells continuously release large amounts of the cytokine IL‐17.^145^, ^146^

Th17 cells are more abundant in patients with SAP than in AP patients. A higher Th17 cell count is frequently linked to a poorer prognosis. According to reports, SAP patients with multiple organ failure had higher circulating Th17 cell counts, which were noticeably higher than those with single organ dysfunction. Compared to individuals who survived SAP, the quantity of Th17 cells in the deceased patients was significantly higher.^147^

Although NK cells and other cells can also release IL‐17, Th17 cells remain the primary producers of the cytokines.^145^ IL‐17 is the executor of the Th17 function. There are six members of the IL‐17 family, numbered IL‐17A through IL‐17F in alphabetical sequence, with IL‐17A having a major proinflammatory function.^148^ Numerous organs, including the gut, lungs, and pancreas, as well as immune cells like macrophages and neutrophils, contain IL‐17A receptors.^146^, ^149^ IL‐17A binds to IL‐17RA in specific organs, leading to damage in those areas. Ni et al.^150^ found that IL‐17A altered the morphology of pancreatic acinar cells, causing changes in vacuolization, basolateral protrusion, and membrane integrity, ultimately resulting in cell necrosis.

Furthermore, in a PADI4‐dependent manner, IL‐17A recruited neutrophils to the site of pancreatic damage. After that, IL17 binds to receptors on the surface of neutrophils, triggering the production of more cytokines and facilitating the maturation and proliferation of neutrophils. Pancreatic cells are damaged due to the increased activation and concentration of ROS and NETs.^151^, ^152^ Additionally, IL‐17A can stabilize IL‐6 mRNA, promote IL‐6 production and release, and enhance SAP's inflammatory cascade by activating the NF‐κB and ERK1/2 MAP kinase pathways in human peripancreatic myofibroblasts.^146^, ^153^

Th17 cell imbalance frequently occurs due to intestinal flora and metabolic issues following the onset of SAP.^154^, ^155^ When Th17 cells are out of balance, they release a high level of IL‐17, which recruits inflammatory cells and collaborates with inflammatory mediators to damage the intestinal mucosa. Through the injured mucosa, bacteria pathogenically transfer into the blood, leading to sepsis. Eventually, this creates a positive feedback loop that exacerbates the illness.^156^

To summarize, Th17 cells play a role in the development of SAP, and IL‐17 is the primary factor responsible for the damage caused by Th17 cells to various organs. Targeted inhibition of Th17 cells and IL‐17 is expected to improve the prognosis of SAP patients.^146^

### 5.5 Tregs

Tregs and related cytokines are much more abundant in SAP patients.^147^ In contrast to Th17 cells, Tregs primarily serve an anti‐inflammatory role in SAP patients, inhibiting excessive immune responses.^157^, ^158^ Human Tregs exhibit different levels of expression for the markers CD45RA and Forkhead box P3 (Foxp3). Miyara et al.^159^ identified human Tregs based on Foxp3 and CD45RA expression: Foxp3^low^ CD45RA^+^ as resting Treg, Foxp3^high^ CD45RA^− ^as activated/effector Treg, and Foxp3^low^ CD45RA^−^ as nonsuppressive cytokine‐producing non‐Treg. Foxp3 is a specific hallmark of Tregs. Although CD25 and CTLA‐4 assist Tregs in carrying out their functions, their specificity is limited.^160^

Tregs can regulate various immune cell functions, limiting an excessive inflammatory response. Tregs release cytokines and express associated antigens that inhibit macrophage differentiation into M1.^161^ The ability of macrophages to respond to proinflammatory stimuli was dramatically reduced when cocultured with Treg cells, indicating clear characteristics of M2 macrophages.^162^, ^163^ Cytokines, including IL‐6, IL‐10, and TNF‐α, are decreased in circulation. There was a notable increase in the expression of the mannose scavenger receptor (CD206) and the hemoglobin scavenger receptor (CD163).

Tregs also regulate DCs. One way that Tregs can prevent DC maturation is by upregulating the expression of CALT‐4. Conversely, Tregs can decrease the expression of DCs' costimulatory molecules CD80 and CD86 while increasing the synthesis of the immunosuppressive enzyme indoleamine 2, 3‐dioxygenase (IDO). The function of effector T cells (Teff) is suppressed by increased IDO.^164^

That being said, Treg suppression might hinder NK cell clearance of microbial infections and tumor cells. Giri et al.^165^ reported that Treg‐mediated suppression of NK cells can lead to systemic vitiligo in patients. Moreover, Tregs can release immunosuppressive molecules such as IL‐10, TGF‐β, and IL‐35 to regulate inflammation.^166^

It is common for SAP patients to have both SIRS and CARS. The two appear concurrently and in no specific order.^107^, ^167^ CARS is a regulatory system that restricts excessive inflammatory responses, and it is primarily controlled by Treg cells and M2‐type macrophages.^168^, ^169^ When CARS occurs, ectopic microorganisms cannot be sufficiently eliminated due to a weakened immune response, which can lead to secondary infections and extra‐pancreatic complications.^170^, ^171^ Circulating FOXP3+/CD25+ Tregs are significantly more abundant in AP mice, with the highest levels observed in infected and necrotic mice, as indicated by research. Furthermore, there is a strong positive relationship between the count of Tregs and the incidence of infection and necrosis in mice.^155^ After the depletion of FOXP3+/CD25+ Tregs was reversed, bacterial infection in mice significantly improved, suggesting that the increased number of activated Tregs promoted secondary infection.^163^, ^172^

AP is a sterile inflammation, whereas SAP is often associated with bacterial infection and pancreatic necrosis. Typical pathogens include *Enterococcus*, *Shigella*, and other gut bacteria. It has been suggested that a change in gut flora is responsible for this phenomenon.^173^ Most of the time, the intestinal mucosal barrier effectively prevents gut bacteria from entering the human circulatory system. Tregs regulate the proliferation of intraepithelial lymphocytes (IELs), which, in turn, modulate the integrity of the intestinal mucosal barrier.^174^ Tregs inhibit the proliferation of IELs, particularly CD8 α+/γδTCR + IELs.^155^ Tregs weaken the intestinal mucosal barrier's defense against infection by inhibiting CD8 α+/γδTCR + IELs, which allows intestinal bacteria to penetrate through the mucosa, leading to bacteremia.^166^

### 5.6 Th17/Treg

There is often a dynamic balance between Th17 cells and Tregs. Individuals may experience multiple illnesses if the two are out of balance.^175^ Researchers observed an increased Th17/Treg subgroup ratio and a significant rise in proinflammatory cytokines in patients with SAP. Dynamic imbalances, such as the Th17/Treg ratio, are often associated with adverse outcomes.^144^, ^147^ It has been demonstrated that high‐volume hemofiltration significantly reduces the Th17/Treg imbalance. Th17/Treg balance improved after 48 h of high‐volume hemofiltration treatment, and patients in the SAP group had a significantly higher survival rate and significantly lower SOFA and APACHE II scores.^147^

### 5.7 Th22 cells

It has now been demonstrated that Th22 cells, a recently discovered subset of CD4^+^ T cells, are linked to a variety of immune‐related illnesses and carcinogenesis. Th22 cells mainly release IL‐22, IL‐17, and TNF‐α.^176^ Due to their remarkable plasticity, Th22 cells can display both Th1 and Th2 cell traits in certain conditions.^177^ Th22 cells depend on IL‐22, a key cytokine, to connect to receptors in nonhematopoietic organs such as the intestine and pancreas.^178^, ^179^

Vasseur et al.^180^ prospectively recruited 62 AP patients and measured their plasma IL‐22 levels. The trial showed that AP patients had higher levels of IL‐22, even if there was a transient reduction in the first few hours. To investigate IL‐22's function in AP, the researchers undertook animal experiments. The research showed that pancreatic and lung injuries in AP mice were significantly lessened following IL‐22 treatment, indicating that the body may use elevated IL‐22 as a defense mechanism in response to damage. Huai et al.^181^ noticed that in mice administered sodium taurocholate‐induced SAP, lung damage was significantly reduced when IL‐22 levels in lung tissue were upregulated.

This protective effect of IL‐22 may be accomplished by increasing the expression of Bcl‐2 and Bcl‐XL, which bind to Beclin‐1 and block the production of autophagic vesicles, hence reducing pancreatitis.^182^ The production of IL‐22 is not limited to Th22 cells, since other immune cells such as Th17 and NK cells can produce it as well.^183^ Consequently, more research is required to understand how Th22 cells relate to lung and pancreatic injury.

### 5.8 T follicular helper (TFH) cells

TFH cells are a unique subset of CD4 + T cells that were identified in 2000. They are mostly located in secondary lymphoid organs, continuously express high levels of programmed cell death protein 1 (PD‐1), and are important for humoral immunity. IL‐21 is one significant cytokine that TFH cells produce. The high level of IL‐21 promotes B lymphocyte maturation and infiltration as well as differentiation. Compared to the MAP group, the SAP group's IL‐21 plasma levels were found to be considerably higher.^184^ There seems to be a connection between IL‐21 and the AP disease process. Further research is necessary to determine the exact role of TFH cells and the impact of IL‐21 in acute pancreatitis, especially in light of IL‐21's potential therapeutic use in modulating the immune response to AP.

### 5.9 T cells in ALI

#### 5.9.1 Th17 cells

SAP patients suffer ALI due to an abnormal accumulation of inflammatory mediators caused by immune dysregulations, as opposed to conventional lung illnesses.^185^, ^186^ In severe circumstances, ARDS could occur, which is directly related to Th17 cells. The ALI mouse model and ARDS patients both exhibited significantly elevated levels of Th17 cells, which seem to be highly activated, along with associated factors IL‐17 and IL‐22.^147^, ^187^, ^188^ Numerous diseases, such as tuberculosis,^189^ chronic obstructive pulmonary diseases,^190^ and asthma,^191^ have been associated with Th17 cells, a type of proinflammatory cell that can irritate airways.

During ALI, neutrophils multiply in the alveolar space and attack lung tissue in significant numbers.^192^ Neutrophils, however, have an extremely short lifespan. Th17 cells help the body continuously recruit neutrophils to the lungs, which is essential for maintaining a high concentration of neutrophils during ALI/ARDS.^193^

Th17 cells are known for producing the proinflammatory cytokine IL‐17. IL‐17 increases inflammatory mediators such as IL‐6, TNF‐α, and colony‐stimulating factors, which recruit and activate neutrophils in the lung tissue, causing lung injury.^146^, ^194^ It is critical to note that neutrophils can induce T cells to differentiate into Th17 cells, which then attract and activate more neutrophils, creating a positive feedback loop that worsens lung injury.^193^, ^195^ Moreover, Th17 cells produce the proinflammatory cytokine IL‐22, which can attract neutrophils to the lungs and exacerbate ALI.^196^, ^197^

Th17 cells' proinflammatory characteristics offer new treatment options for patients with ALI. Some studies have shown that rapamycin inhibits the development of Th17 cells, thereby significantly reducing lung injury in mice.^197^ Through the Notch signaling pathway, protein kinase C theta (PKCθ) can suppress Th17 cells, hence reducing lung neutrophil infiltration.^198^

#### 5.9.2 Tregs

A growing number of investigations have explored the involvement of CD4 + CD25 + FOXP3+ Treg cells in ALI in recent years. According to research, in mice and humans with ALI, the recruitment of CD4 + CD25 + FOXP3+ Treg cells in the alveoli, particularly through the leukotriene B4(LTB4)‐leukotriene B4 receptor (BLT1) pathway,^199^ could decrease lung fibroproliferation^200^ and improve lung inflammation.^201^

The count of activated CD4 + CD25 + FOXP3+ Treg cells skyrocketed during ALI. Adamzik et al.^202^ concluded that both the ARDS death group and the ARDS survival group had significantly more alveolar CD4 + CD25 + FOXP3+ Treg cells than the normal control group (*p* < .05). The number of CD4 + CD25 + FOXP3+ Treg cells is positively correlated with the severity of ALI and can be used to predict the prognosis of a patient with ALI.^203^

The accumulation of excessive amounts of inflammatory mediators in the lungs is the primary cause of ALI. Patients with ALI frequently suffer from pneumonia and hypoxemia due to diffuse alveolar damage caused by uncontrolled neutrophils, macrophages, and other proinflammatory factors. CD4 + CD25 + FOXP3+ Treg cells are now significantly contributing to the alleviation of pulmonary symptoms in ALI patients through a variety of mechanisms.^204^

In ALI patients, CD4 + CD25 + FOXP3+ Treg cells have the potential to decrease pulmonary inflammation by reducing the influx of inflammatory cells into the lungs. The activity of neutrophils is also inhibited, and apoptosis is promoted by CD4 + CD25 + FOXP3+ Treg cells.^205^ Moreover, CD4 + CD25 + FOXP3+ Treg cells release TGF‐β, IL‐10, IL‐35, and other substances, which restrict Th17 cell and macrophage maturation.^166^, ^203^, ^204^

In the course of ALI, CD4 + CD25 + FOXP3+ Treg cells also function as “repairers.”^187^ Amphiregulin and keratinocyte growth factor (kgf) are primarily responsible for this impact.^206^, ^207^ Amphiregulin, which is produced by CD4 + CD25 + FOXP3+ Treg cells, contributes to healing inflammatory injuries that are both membrane‐bound and cleaved.^206^

Alveolar epithelial type II cells (AE2) can be stimulated to proliferate more quickly by CD4 + CD25 + FOXP3+ Treg cells in response to damage.^207^ The primary element involved in alveolar epithelial cell repair is AE2 cells. Cell proliferation and transdifferentiation occur in response to injury to alveolar epithelial type I cells (AE1) to maintain the integrity of the epithelial tissue. AE2 cells proliferate at the site of the initial injury.^208^ In vitro, AE2 cells were cocultured with wild‐type Treg cells and Treg cells deficient in Kgf. Researchers discovered that AE2 cells cocultured with wild‐type Treg cells proliferated rapidly.^207^ The kgf, which is primarily produced by Tregs, can significantly enhance the proliferation rate of AE2 cells while also repairing the injured epithelium.^209^

The late stages of ALI are characterized by fibroplasia, a natural process of recovery in which fibroblasts accumulate in alveolar cells and release abundant amounts of collagen. Respiratory fibrosis begins when proliferation gets out of control. CXCL12 is a mediator that stimulates fibroblast recruitment and contributes to pulmonary fibrosis. Research has shown that Tregs can decrease lung levels of CXCL12 and inhibit collagen deposition and fibroblast recruitment via the CXCL12‐CXCR4 pathway, thus alleviating pulmonary fibrosis.^201^

#### 5.9.3 Th17/Treg

Th17 and Treg cells follow the same developmental pathway and are both regulated by TGF‐β1. TGF‐β1 regulates the differentiation of CD4 + T cells into Th17 or Treg cells.^210^ When TCRs are activated, TGF‐β1 increases Foxp3 gene expression and converts CD4 + CD25− Treg cells into CD4 + CD25+Foxp3+ Treg cells.^211^, ^212^, ^213^ Researchers have found that Fxop3 is aberrantly expressed in several inflammatory disorders, and Treg cells can transform into Th17 cells.^214^ According to earlier research by Park et al.,^215^ IL‐33 may use DCs to convert CD25^hi^ Tregs into Th17 cells.

Normally, the quantity of Th17 cells and Tregs fluctuates dynamically, while the ratio of Th17/Treg maintains a dynamic balance. Patients with SAP have poor outcomes due to the disruption of the dynamic balance between Th17 cells and Tregs, Tregs transform into Th17 cells, leading to the disappearance of their mutual antagonism. This was established using a prospective experiment. ARDS patients with a Th17/Treg ratio >0.79 exhibited a worse prognosis and a higher mortality rate (*p* < .001) after 28 days.^188^ The area under the ROC curve (AUC) of the Th17/Treg ratio for predicting 28‐day death in ARDS patients was up to 0.824 (95% CI 0.722−0.901). Th17/Treg ratio can be utilized to predict the prognosis of SAP‐ALI patients.

## 6 MYELOID‐DERIVED SUPPRESSOR CELLS (MDSCS)

MDSCs are immature bone marrow‐derived cells with immunosuppressive qualities and the markers CD33^+^, CD11b^+^, and HLA^‐^DR^‐/low^. Due to differences in their behavior and morphology, researchers have separated MDSCs into two subpopulations: CD14^+^ monocytic MDSCs (M‐MDSCs) and CD66b^+^ granulocytic MDSCs (G‐MDSCs).^216^ Existing research has shown that MDSCs' immunosuppressive function increases the growth and metastasis of malignant tumor cells, such as breast, rectal, and liver malignancies, and contributes to the failure of radiotherapy/chemotherapy in patients.^217^ Researchers are curious about MDSCs' immunosuppressive properties: do MDSCs converse with other immune cells during the SAP process, thereby exacerbating the severity of the disease?

Ding et al.^218^ investigated MDSCs in the peripheral blood of HCs, MAP patients, and SAP patients. The results showed that the percentage of MDSCs in PBMC was significantly higher in the SAP group (20.90 ± 13.580%) than in the MAP group (11.40 ± 6.698%) and HCs (1.74 ± 0.9780%) (*p* < .01). Additionally, the peripheral blood of patients in both the MAP and SAP groups had significantly higher levels of MDSCs than HCs. The degree of AP illness was strongly connected with the amount of MDSCs, particularly M‐MDSCs.

During the experiment, the researchers revealed that MDSCs in the SAP group had significantly higher immunosuppressive activity than the HCs group. When CD3^+^ T cells were cultivated with MDSCs in the SAP and HCs groups, respectively, the SAP group's value‐added rate of CD3^+^ T cells (61.77 ± 2.076%) was significantly lower than that of the HC group (80.33 ± 3.117%) (*p* < .05). According to the study mentioned above, when SAP developed, the number of MDSCs grew along with the disease's severity, and T cells' capacity to prevent growth also improved.

Arginase plays a major role in the inhibitory action of MDSCs on T cells. Arginase is abundantly expressed and active in MDSCs, resulting in enhanced arginine catabolism and a circulating arginine deficit.^219^ Arginine, the most versatile amino acid, is vital in controlling T cell proliferation and other processes.^220^ Depletion of arginine in the peripheral circulation results in decreased expression of CD3ζ, loss of T‐cell receptor signaling, and inhibition of T‐cell proliferation.^221^, ^222^ Taheri et al.'s in vitro findings^223^ showed that there was a decrease in cytokines, a reduction in CD3ζ expression, and an inhibition of cell proliferation when the circulating arginine concentration fell below 60 μmol/L. Furthermore, within MDSCs, ROS expression was markedly elevated.

Unfortunately, the immunosuppressive actions of MDSCs initially regulate the enhanced inflammatory response in SIRS. However, the immunosuppressive effect of MDSCs increases with infection severity, which can result in CRAS, secondary infections, and eventually multiorgan damage.^224^

## 7 POTENTIAL TREATMENT STRATEGIES AND FUTURE DIRECTIONS

Further challenges to immunotherapy research stem from the intricacies of the immune response during SAP. Targeted therapy aimed at immune cells is one possible SAP treatment choice. The immunosuppressant FTY720 reduced CD4+ and CD8^+^ T cell infiltration and targeted and suppressed Th cells, which in turn alleviated the severity of SAP and late pancreatic fibrosis.^225^, ^226^ A selective type I SIP receptor agonist, SEW2871, reduces the number of CD4^+^ T lymphocytes in the pancreas and depletes them in peripheral organs, thereby suppressing the inflammatory response.^227^ Tacrolimus, a prominent immunosuppressant, inhibits apoptosis and T lymphocyte infiltration in the pancreas, potentially preventing the development of CP.^228^ Researchers found that nicotine stimulation at a level of 100−300 μg/kg increased the number and repression of Tregs in SAP and improved the prognosis of SAP mice induced with sodium taurocholate. It also stimulated the production of immunomodulatory molecules and the secretion of TGF‐β1.^169^ Additionally, it has been shown that thymidine inhibits the release of cytokines by controlling the development of CD3/CD4^+^ T cells.^229^ The introduction of immunomodulatory medications has provided new therapy choices for more SAP patients. However, further investigation is needed to evaluate the effectiveness of these immunomodulatory therapies.

It's important to note that human immunity is complex. Targeted therapy targeting one immune cell may affect the behavior of other immune cells. As a result, personalized immunomodulatory therapy is essential. Before commencing immunomodulatory therapy, a thorough evaluation of the patient's condition, underlying conditions, and immunological status should be performed to identify the proper use of relevant medicines.

Future studies will reveal the role of immune cells in SAP and ALI, identify effective treatment targets, and obtain precise “guidance.” Concurrently, an all‐encompassing and methodical immune assessment framework must be constructed to bolster immunomodulation treatment and reduce the rates of SAP patient death.

## 8 CONCLUSION

In this review, we focus on the intricate roles that various immune cells play in the development of ALI and SAP, suggesting a new direction for the treatment and prognosis of SAP and related ALI patients. However, more research is needed to fully comprehend the intricate mechanism underlying the synergistic effect of cytokines and immune cells. Ultimately, a deeper comprehension of immune cells will enable clinicians to create more personalized therapy programs in the future to address the immunological imbalance in SAP and ALI patients at an early stage of the disease, thus enhancing their prognosis.

## AUTHOR CONTRIBUTIONS

Qi Liu wrote the main manuscript, Xiaomei Zhu designed the figures, and Shubin Guo reviewed the manuscript and figures.

## CONFLICT OF INTEREST STATEMENT

The authors declare no conflict of interest.
